# Body Loading during an Intensive Yoga Exercise Routine and a Cycle Ergometer Test

**DOI:** 10.3390/ijerph20054157

**Published:** 2023-02-25

**Authors:** Krzysztof Stec, Karol Pilis, Wiesław Pilis, Przemysław Miodek, Anna Pilis, Sławomir Letkiewicz

**Affiliations:** 1 Department of Health Sciences, Jan Długosz University in Częstochowa, 42-200 Częstochowa, Poland; 2 Institute of Health Prophylaxis, 42-200 Częstochowa, Poland

**Keywords:** yoga, cardiovascular reactivity, metabolism, pulmonary reactivity, physical fitness

## Abstract

The present study compared the effects on the cardiovascular, respiratory, and metabolic functions of the practice of an intensive yoga exercise routine called Dynamic Suryanamaskar (DSN) and a cycle ergometer test (CET) of increasing intensity. The study involved 18 middle-aged volunteers who had previously practiced DSN. The study was conducted in two series (i.e., as CET and DSN with similar intensity) until complete exhaustion. At rest (R), at the ventilatory anaerobic threshold (VAT), and at the maximum workload (ML), the variables characterizing cardiovascular, respiratory, and metabolic functions were determined. In addition, the subjective intensity of both efforts was determined using the Borg test. No functional differences were observed in the cardiovascular, respiratory, and metabolic systems at similar CET and DSN intensities. Respondents experienced less subjective workload during DSN than during CET (*p* < 0.001). Since DSN intensifies the activity of the cardiovascular, respiratory, and metabolic systems to a similar degree to CET both at VAT and ML, but causes less subjective fatigue, this yogic practice can be used as a laboratory exercise test and as an effective training medium.

## 1. Introduction

Among the laboratory tests available to evaluate the human capacity for physical exercise, the most frequently used method is to load the lower limbs by means of a cycle ergometer or a mechanical treadmill (walking or running). Other laboratory methods include rhythmic step-climbing [1], running up stairs [2], isometric forearm squeeze [3], performing squats with the weight of one’s own body [4], maximum jump test in the standing position [4,5,6], or a series of vertical jumps [7]. In tests performed outside the laboratory, walking [8,9], running, swimming, cycling [10,11], running with turns on designated tracks [12,13], and many other methods have also been used to provide an exercise load. All of the above-mentioned tests have limitations in their ability to enable the assessment of exercise capacity, thus limiting the scope for their use. These limitations include maximum and supramaximal exercise intensity [14], excessive loading of a specific muscle group [1,3,14], the risk of bodily injury [2], the state of health and general condition of the test participants [8,9], and, in some cases, the weather conditions when performing the tests [10,11].

The hypothesis to be tested in the most general sense (this is defined more precisely below) is that these limitations can be overcome to a significant degree by using the yoga practice known as Suryanamaskar (SN) as the exercise load. SN is part of hatha or physical yogic practice. It consists of an uninterrupted sequence of asanas, usually 12 in number, beginning and ending in a standing position, and performed with the maximum possible forward and backward extension of the body in the sagittal plane, along with deep breathing aligned to the movements. Completing the sequence of 12 asanas constitutes one round of the practice. Two consecutive rounds, each beginning with a backward stretch from a standing position and involving an alternate stretching backward of each leg, form one SN cycle [15,16]. These postures (asanas) are coordinated with controlled breathing (pranayama), focused attention (dharana), and sometimes, the silent chanting of mantras. Various yoga schools employ many technical modifications to SN. The basic version of SN, as described in the Bihar School of Yoga (BSY) tradition, consists of 12 positions [17]. The Krishnamacharya Vinyasa Yoga tradition has 13 positions [18]. The tradition of the Shivananda Yoga Vedanta Center is similar to that of the BSY [19]. The Swami Vivekananda Kendra tradition also has 12 positions, and is similar to that of the BSY [20].

It has been shown that the rate of SN performance has a significant adaptive effect on the body [8]. Among the many adaptive effects, SN practice has a positive effect on health, increases physical performance [21], improves insulin resistance, glucose tolerance, and positively modifies the serum lipid profile [22,23,24]. SN practice also improves the functioning of the internal organs of the abdominal cavity and of the skin as well as increasing the body’s immunity [25] and improving the cardiopulmonary system of both the healthy and the sick [26,27]. Moreover, SN practice has been shown to optimize body composition [28], metabolism [29], endocrine functions [30], and the mental functioning of practitioners [31].

SN as an exercise load is a very convenient, safe, and inexpensive exercise test that can be performed in almost any conditions. It has the advantage that it does not require the use of any equipment or apparatus (only about 2 square meters of flat floor are needed). It can be performed by men, women, children, adults, and the elderly. It loads many muscle groups simultaneously, and its pace of performance can be adjusted to the capacity of the tested person.

However, SN as an exercise load has not hitherto always been defined in a precise way for the purpose of the various published studies in which it has been used, though some exceptions to this structure are discussed below. SN can be performed in a manner that is very slow, slow, medium, fast, or very fast and, as the speed of the exercise increases, so does the intensity. So far, the effects of using the slow, medium, and fast versions of SN have been partially characterized. In the ‘fast’ or ‘rapid’ manner, Bhavanani et al. [32] required that all 12 postures had to be completed in 2 min, and, therefore, 15 rounds were performed in 30 to 40 min. On the other hand, in the ‘slow’ manner, Bhavanani et al. [32] stipulated that each of the 12 postures was held for 30 s, and therefore, each round took 6 min to complete and five rounds were performed in 30 to 40 min. In comparison, the rate of performance of the DSN version was about 16 times higher than the ‘fast’ rate of Bhavanani et al., that is, a round took 7.5 s compared to 120. Unfortunately, very few SN studies have provided a clear and unequivocal definition of the SN style used and a statement of the intensity (speed) of performance achieved. For example, DSN is performed at a speed of eight rounds per minute (7.5 s × 8).

Only one study has shown that SN has a positive effect on the physiological variables, which applies to pulmonary functions, respiratory pressures, handgrip strength, and endurance as well as the resting cardiovascular parameters. It was also found that the slow version of SN in its adaptive effects was similar to the impact of traditional postural yoga, while the medium and fast versions had similar effects in the body to increasingly intense aerobic exercise [32,33]. The impact of SN and the cycle ergometer exercise test on the body has also been assessed in a published study. Unfortunately, these comparisons concerned only the exercise intensity in relation to VO_2max_ at the levels of 10–20%, 21–40%, and 41–50% [33].

In order to obtain a better understanding of the effects of the slow and fast versions of SN on the body, Stec decided to describe in more detail the techniques of performing the practice. The version chosen consisted of 12 asanas, and was based on the sequence known as the Rishikesh Series. Detailed descriptions are to be found in his book published by the SVYASA Yoga University [16]. The technique has four levels. The first three consist of very slow, slow, and medium speeds of practice, along with breath control, focusing and fixing of the mind, and, at the third level, the chanting or repeating of traditional mantras. The practice conducted at the fourth highest level is very fast. It requires that, depending on the individual’s capacity, about 40 rounds of SN will be performed in 5 min, which requires the performance of one round in 7.5 to 8 s. It was this exceptionally fast pace and intensity of the practice that suggested the addition of the term ‘Dynamic’ to the normal appellation, ‘Suryanamaskar’. Additional support for the use of this original name can be found in the capabilities of the best adepts, who can perform 1000 rounds of DSN continuously at the fastest rate. In the time required, of just over 2 h, a man of 80 kg in weight can lose about 4 kg body mass. This degree of effort is extremely aerobic. At this level, the practitioner is focused on maintaining the highest pace and on the flow of the breath. In such a state, between one and four breaths will be taken per round instead of the usual six. Another study by Stec et al. [34] showed that energy expenditure during the practice of DSN was high and similar to that found during the maximum intensity cycle ergometer test, that is to say, it reached 14.5–16 METs (about 95–100% VO_2max_). Unfortunately, the small number of participants in this trial (three) means that this observation was obtained from what can only be considered as a case study, that is, with the result being no more than indicative. Thus, there is currently no significant published research that would definitively demonstrate the physiological effects of DSN practice at the maximum level.

Furthermore, in not merely Western societies but also elsewhere, a tendency can be found to adopt modified forms of yoga practice, that is, that are less complete, and so less demanding. For example, such a modification was made to SN practice by Iyengar [35]. The leading role in that form of practice is played by asanas, while much less attention is paid to breath control, mental concentration, and the repetition of mantras. Additionally, in the Iyengar style, the SN form is quite different from that found in the Rishikesh Series form used in DSN. The lack of uniformity in the practice of SN is thus an additional factor making the comparison of results in published studies less secure.

As a result of the foregoing factors, we decided to conduct an experiment in which the physiological effects of a closely defined and highly controlled DSN practice and that of the maximum intensity cycle ergometer test (CET) were compared. It was considered that despite the fact that the loads used in both tests differed in the nature of the work performed, in a soundly designed study, the results would be sufficiently robust to begin the process of constructing a reliable body of work comparing the results of DSN with other forms of aerobic exercise.

### Hypotheses

Assuming that during both DSN and CET the physiological load may reach its maximum values, this study compared the reactions of the circulatory and respiratory systems of a mixed group of men and women, advanced in DSN and CET techniques, together with the participants’ subjective assessment of the exercise load during both trials.

In order to achieve this goal, two research hypotheses were put forward:

**Hypothesis 1.**  *The load on the body by DSN and CET is similar and reaches maximum values*.

**Hypothesis 2.**  *Performing the DSN test causes subjectively less fatigue than the CET test*.

## 2. Materials and Methods

### 2.1. Study Design

The study consisted of two exercise tests in which 18 people participated. In the first, the ergometric exercise test (CET) was performed, and, in the second, with the same people participating, the exercise load was provided by DSN. Since both types of effort differ significantly in the nature and technique of the work performed, in order to avoid any obstructive effect of having to learn the technique of the movements, before the main examinations, all participants familiarized themselves with the ergometric test (CET) and with DSN, with complete connection to the testing equipment, on two separate occasions, on the sixth and third days before the first CET trial. In addition, in order to avoid measurement errors during the DSN, the medical staff assigned to carry out the supporting technical work during the actual tests, performed all the work of connecting the wires linking the persons under test with the equipment.

The results of the registered variables of the circulatory system, respiration, and metabolic indices obtained during each set of tests were compared with one another in order to assess the extent to which these different kinds of effort strain the body.

**Figure 1:**
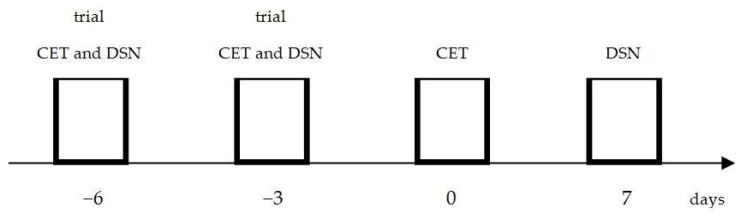
Scheme of the trial and basic exercise tests.

Figure 1 shows the outline of the research.

### 2.2. Subjects

Eighteen middle-aged (53.28 ± 7.31 years) volunteers (seven women and 11 men) participated in the study. These participants had many years of experience in competitive sports or yoga practice. Their training experience in practicing DSN averaged 2.14 ± 0.82 years. They were healthy people who did not drink alcohol or smoke cigarettes, and who were not concurrently with the study taking any medications, stimulants, or drugs. As a matter of administrative importance, attention must be drawn to the great difficulty associated with ensuring the participation in the study of volunteers with adequate prior experience in DSN practice. This difficulty arose from the fact that the DSN form of practice is not widely undertaken in the yoga practitioner community. This fact resulted in the relatively small number of people being able to participate.

### 2.3. Procedures

The participants came to the laboratory in the morning between 8:00 am and 9:30 am, after 12 h of being in a postprandial state. In the first stage of the study, age, body height (BH), and certain somatic variables were recorded. The latter included body mass (BM), body fat (FAT), total body water (TBW), fat-free mass (FFM), and body mass index (BMI), which were determined using a body composition analyzer (Tanita BS 418-MA). The subjects then undertook the CET. After 7 days, DSN exercise was undertaken.

Each round of the DSN consisted of 12 consecutive asanas, as described in the Rishikesh Series [16]: Pranamasana, Hasta Utthanasana, Padahastanasana, Ashwa Sanchalanasana, Parvatasana, Ashtanga Namaskara, Bhujangasana, Parvatasana, Ashwa Sanchalanasana, Padahastanasana, Hasta Utthanasana, Pranamasana. The described version of the DSN is depicted in Appendix A. Each round began and ended with the prayer posture (Pranamasana). The next round began with the opposite leg stretching backward. Two consecutive rounds constituted one DSN cycle. Due to the individually determined maximum pace of performing DSN, the subjects did not maintain coordinated breathing while performing the asanas in a smooth and continuous fashion.

Before each test, CET or DSN, the face mask was tightly fastened and connected to a respiratory analyzer (Ergo Card, Belgium), and the HR transmitter (Polar) was placed on the chest of the subject. The frequency of work, that is, the rate of pedaling, during CET was 60 rpm, with an initial load of 30 W, which was systematically increased every 3 min by 30 W, until the maximum individual fatigue (refusal point) was achieved. At rest and throughout the duration of CET and DSN, the following variables were recorded: heart rate (HR), pulmonary ventilation (V_E_), oxygen uptake (VO_2_), carbon dioxide excretion (VCO_2_), respiratory exchange ratio (RER), cardiac output (CO), and stroke volume (SV). In addition, the following variables were calculated: ventilatory equivalents of O_2_ (EQO_2_, i.e., V_E_/VO_2_), ventilatory equivalents of CO_2_ (EQCO_2_, i.e., V_E_/VCO_2_), energy expenditure (EE), and VO_2_/HR ratio (oxygen pulse). In the case of CET, the maximum power (ML) was also recorded, and in DSN, the number of cycles performed was counted. The speed of DSN execution was regulated by each of the subjects, so that in a similar time to that in which the CET had been performed, the state of maximum fatigue was achieved by performing as many cycles as possible. The size of the current load during DSN was assessed by the heart rate recorded in the continuous monitoring system. The DSN was terminated when the subject refused further exercise due to fatigue or reached the maximum HR recorded during the previously performed CET. In order to assess the subjective intensity of exercise achieved during CET and DSN, the Borg test was performed [36].

Using the values of pulmonary ventilation, EQO2, EQCO2, and their changes resulting from successive increases in workload as the CET and DSN were continued, the ventilatory anaerobic threshold (VAT) values were determined as the break points of these curves [37].

Minute’s energy expenditure (EE) was calculated according to the modified Weir equation [38]:

- EE = (3.94 × VO_2_) + (1.1 × VCO_2_), where EE is the energy expenditure (kcal·min^−1^), VO_2_ is the size of the oxygen uptake (l*min^−1^), and VCO_2_ is the volume of carbon dioxide excretion (L·min^−1^).
- Cardiac output (CO) using the quick breath analyzer Ergo Card was calculated from the Fick formula, based on the non-invasive method for determining the amount of oxygen absorbed by the body [39].

The Bioethics Research Committee of Jan Dlugosz University in Czestochowa, Poland gave its approval for the present study. Study procedures were described in detail and written informed consent was obtained from all participants.

### 2.4. Statistical Analysis

Basic statistical analyses of all variables were performed and the mean ± SD values were calculated. The Shapiro–Wilk test was used to check the normality of the distribution. Since all variables had non-normal distributions, non-parametric statistics were used in further calculations. Friedman repeated measures analysis of variance by ranks with the Dunn–Bonferroni post hoc test was used to show differences between the results recorded during CET and DSN. The paired Wilcoxon test was used to compare the results of the Borg test recorded in CET and DSN. The Mann–Whitney U test was used to determine the sex differences of the analyzed variables. Values at *p* < 0.05 were considered statistically significant. All statistical analyses were performed using the STATISTICA software (Dell Statistica—data analysis software system, version 13.3).

## 3. Results

The somatic data of the volunteers under study indicated that their body structures were within the normal physiological limits (Table 1).

The Friedman analysis of variance showed that the main effect on the intergroup and intragroup differences was statistically significant for all of the analyzed functional variables (*p* < 0.001) listed in Table 2 and Table 3. However, the values of all variables recorded at R, VAT, and ML were similar and did not differ statistically between CET and DSN. The post hoc analysis showed that the applied physical effort resulted in a statistically significant increase in the values of V_E_, VO_2_, VCO_2_, RER, EE (Table 2), and HR, SV, CO, VO_2_/HR (Table 3) marked at VAT and ML in comparison to the values occurring at R in both the CET and DSN tests. Only the EQCO_2_ values determined at VAT were significantly lower than at R in relation to the DSN (*p* < 0.01) and CET (*p* < 0.001). In addition, it was shown that in the DSN test, higher EQCO_2_ and HR (*p* < 0.05) and EQO_2_ (*p* < 0.01) values were achieved at ML than at VAT. During CET under ML conditions, higher values of V_E_, relative VO_2_, EQCO_2_, EE, HR, VO_2_/HR (*p* < 0.05), VCO_2_ (*p* < 0.01), and EQO_2_ (*p* < 0.001) were recorded than at VAT. Table 4 and Table 5 present the values of the analyzed variables together with the determination of gender differences. However, no deeper analyses were conducted in this regard as the gender differences were not the aim of the presented work.

VAT during CET was obtained on average in 16.33 ± 5.17 min at a load of 163.33 ± 51.68 W, oxygen consumption of 30.89 ± 8.68 mL·min^−1^·kg^−1^ (76.74 ± 8.31% VO_2max_), which generated EE 8.83 ± 2.48 MET at a heart rate of 87.65 ± 4.38% HRmax. The CET lasted 20.67 ± 5.43 min, and the ML achieved in this test was 206.67 ± 54.34 W, with a VO_2max_ of 40.22 ± 10.25 mL·min^−1^·kg^−1^, which gave an EE of 11.49 ± 2.93 MET. VAT was observed after 6.13 ± 0.7 min DSN with oxygen uptake of 32.22 ± 7.28 mL·min^−1^·kg^−1^ (82.73 ± 4.88% VO_2max_), which generated an EE of 9.21 ± 2.07 MET) and HR of 89.12 ± 5.61% HRmax. During 19.91 ± 5.23 min of DSN, the subjects performed 59.72 ± 3.91 cycles (i.e., 2.91 ± 1.21 cycles per minute). The final stages of this test generated an oxygen consumption of 38.50 ± 7.06 mL·min^−1^·kg^−1^ and resulted in an EE of 11.03 ± 2.02 MET.

It was also shown that subjective exercise intensity assessed by the subjects was lower during DSN than CET (*p* < 0.001), and was at the level of 15.72 ± 1.41 and 18.61 ± 0.61 points on the Borg scale, respectively.

## 4. Discussion

Since all subjects participating in this study were healthy people without any medical contraindications, the recorded variables at the final CET load were taken as the maximum values. Therefore, since all variables recorded at VAT and ML in the CET and DSN tests relating to circulatory and respiratory functions did not differ statistically, it should be assumed that the DSN test was also the maximum burden for the body. The high EE values achieved in both tests at the final load, exceeding 11.00 MET, were classified as close to maximum or maximum [40]. Data from the literature also indicate that the amount of energy expenditure during the fast form of SN is defined as high or very high, similar to other aerobic exercises [41,42]. Additionally, the HR levels achieved at the final load levels in both tests indicate that these were the maximum values that occurred in people of about 50 years of age, and therefore, the final power that was achieved should be considered as the maximum.

The physical capacity of the participants in the studies, determined on the basis of VO_2max_, should be assessed as high for this age group [43]. Another reliable indicator for assessing physical capacity is the amount of oxygen uptake by the body, defined by VAT and expressed as a percentage of the maximum oxygen uptake. In untrained people, this value is at the level of 50% of VO_2max_. In our studies, this indicator exceeded 76% of VO2max in the case of CET and over 82% of VO_2max_ in the case of DSN. This result provides irrefutable proof of the high physical capacity of the people participating in some prior studies [44,45,46], and demonstrates that the DSN test meets all of the criteria needed to demonstrate its suitability for use in the assessment of human exercise capacity, similarly to the standardized CET test.

The fact of there being a similar effect of both exercise loads on the body was also confirmed by the reports of Sinha et al. [33], who found a similar load on the cardiovascular system during the performance of SN and an ergometer exercise test at varying intensities of very low, low, and medium [47]. It is also significant that the participants in the current study reported a significantly lower value of subjective workload in the Borg test during DSN than during the CET (*p* < 0.001). This means that performing DSN provided the subjects with a greater degree of comfort than that during the prior CET exercise. During the CET exercise, there was a large overload on the lower limbs without much involvement of the trunk and upper limb muscles, as a result of which the study participants complained of severe muscle pain in the legs, which in some subjects resulted in their being unable to continue. During DSN, on the other hand, the effort consisted in alternating contractions and maximal stretches of the upper and lower limbs as well as the front and back muscles of the trunk, and therefore there were no local muscle pains and the body became more flexible [48]. Since the practice of SN leads to the activation of the parasympathetic nervous system [40], and the DSN has many features in common with that practice, it should be expected that, since the volunteers participating in the experiment had 2.14 ± 0.82 years of experience in this field, then, during the performance of the DSN, the parasympathetic tone would be greater than during CET. It is also known that increased parasympathetic tone accompanies relaxation [49]. It is, therefore, legitimate to suggest that the better subjectively reported exercise tolerance during the DSN than in the CET, apart from the versatile and alternating muscle work, could have been based on a greater state of relaxation caused by the increased tone of the parasympathetic system. Unfortunately, the vegetative responses of the organism were not recorded during this series of studies, so this line of reasoning remains largely hypothetical and is a topic that should be investigated in future studies.

The functional effects described underlie the improvement in the respiratory and circulatory system functions [50]. In the present study, DSN did not cause functional changes in the cardio-respiratory system that were significantly different from those that were recorded during CET.

DSN, apart from its usefulness as a test to assess physical and physiological capacity, can be used as a training method. It is also suggested that SN is a better training method than almost all others in the following regards, namely, that it does not require any tools or prior organization and needs little space, thus providing benefits of both economy and time [9].

In summary, it can be stated that the applied effort of DSN caused a similar load on the circulatory and respiratory systems and an intensified metabolic effect similar to those found in the ergometer exercise test at the submaximal and maximum intensities, and that the subjects felt more comfortable during the yogic practice than during the CET. Therefore, it should be recognized that DSN may be as useful in loading the body with physical effort as a standardized ergometer exercise test.

As a result of the existence of a convenient form of burdening the body with physical effort such as DSN practice, it is appropriate to consider developing a physical fitness test in which the involvement of muscle mass will be greater than during the CET test. Before constructing such a test, it would be important to compare the reactions of the circulatory and respiratory systems at maximum running load on a mechanical treadmill and during DSN. In both of these tests, it seems likely that the load on the cardiovascular system would be similar to and perhaps greater than that experienced during the CET test, which activates less muscle mass during exercise than the running test or DSN. It should, however, be kept in mind that one difficulty in developing such a test is likely to be the technical intricacies of executing the DSN practice at the required level of proficiency. While such technical difficulties would be likely to reduce the range of applicability of such a test, nonetheless, despite its narrower application, it would be a useful specific exercise test for people who have had previous contact with traditional yoga or its various modifications.

## 5. Conclusions

Different body loads can be achieved during DSN including the maximum, depending on the intensity and duration of this yoga practice. It should be noted that during DSN, participants reported experiencing less fatigue than during CET. This has been attributed to the loading of most muscles and joints equally. The workload on the cardiovascular, respiratory, and metabolic systems during DSN was similar to that during CET, and it should therefore be concluded that DSN can be used as a laboratory exercise test. In addition, due to the lack of any requirement for apparatus and to the possibility of performing this form of exercise anywhere that there is the most minimal amount of space, DSN can also be considered to be a most effective universal physical training medium.

## Figures and Tables

**Table 1:** Somatic characteristics (mean ± SD) of the whole group of subjects (W + M; n = 18), group of women (W; n = 7), group of men (M; n = 11).

Group/*p*	AGE [Years]	BM [kg]	BH [cm]	BMI [kg·m^−2^]	FAT [%]	FFM [kg]	TBW [kg]
W + M	53.28 7.31	69.41 10.14	175.44 10.02	22.49 2.14	20.31 6.88	55.71 11.50	40.78 8.42
W	51.43 7.25	59.56 4.82	167.14 8.07	21.43 2.57	27.51 4.11	43.09 3.07	31.54 2.26
M	54.45 7.45	75.67 7.05	180.73 7.27	23.17 1.60	15.73 3.30	63.75 6.06	46.66 4.44
p*	0.479	0.001	0.001	0.085	0.001	0.001	0.001

BM—body mass; BH—body height; BMI—body mass index; FAT—body fat; FFM—fat free mass; TBW—total body water; *p*—statistical differences between W and M; p* U Mann–Whitney test.

**Table 2:** Statistical comparisons of the respiratory and metabolic values obtained during DSN and CET (n = 18).

Variables	Group	R					VAT					ML					*p* *
		Mean	±SD	M	MIN	MAX	Mean	±SD	M	MIN	MAX	Mean	±SD	M	MIN	MAX	Main Effect
V_E_ [L·min^−1^]	DSN	11.24	3.34 ^aa^	11.15	7.50	18.80	61.27	18.94	60.75	23.90	92.20	88.77	31.31 ^bbb^	82.55	44.90	135.00	<0.001
	CET	11.91	2.85 ^d^	11.65	7.40	18.40	60.23	18.88 ^f^	65.15	28.30	87.70	100.69	36.18 ^eee^	90.95	41.00	165.70	
VO_2_ [L·min^−1^]	DSN	0.39	0.09 ^aa^	0.38	0.26	0.63	2.24	0.60	2.28	1.32	3.38	2.71	0.73 ^bbb^	2.72	1.66	4.26	<0.001
	CET	0.41	0.14 ^d^	0.39	0.24	0.62	2.18	0.74	2.13	1.01	3.43	2.82	0.88 ^eee^	2.73	1.30	4.63	
VCO_2_ [L·min^−1^]	DSN	0.32	0.07 ^aa^	0.31	0.23	0.46	2.20	0.66	2.14	1.03	3.40	2.80	0.83 ^bbb^	2.94	1.32	4.39	<0.001
	CET	0.34	0.08 ^d^	0.34	0.20	0.53	2.20	0.67 ^ff^	2.25	1.21	3.19	3.27	0.81 ^eee^	3.14	2.02	4.58	
RER	DSN	0.85	0.06 ^a^	0.83	0.73	0.96	0.98	0.08	0.99	0.85	1.09	1.03	0.10 ^bbb^	1.04	0.90	1.20	<0.001
	CET	0.85	0.08 ^dd^	0.85	0.72	0.97	1.03	0.12	1.00	0.85	1.15	1.19	0.18 ^eee^	1.17	0.99	1.49	
VO_2_ [mL·min^−1·^kg^−1^]	DSN	5.50	1.34 ^aa^	5.00	3.00	9.00	32.22	7.28	32.50	15.00	45.00	38.50	7.06 ^bbb^	37.50	25.00	50.00	<0.001
	CET	6.00	2.20 ^d^	5.50	4.00	11.00	30.89	8.68 ^f^	28.00	16.00	43.00	40.22	10.25 ^eee^	37.50	20.00	57.00	
EQO_2_	DSN	29.30	5.74	29.03	19.38	46.92	27.30	5.06 ^cc^	27.93	17.45	37.35	32.44	6.79	32.01	21.02	45.53	<0.001
	CET	29.73	4.51	30.99	22.44	39.49	28.28	5.25 ^fff^	26.20	22.51	38.51	37.22	10.91	36.51	27.62	52.62	
EQCO_2_	DSN	34.77	5.52 ^aa^	34.43	24.47	48.16	27.88	4.03 ^c^	28.95	22.00	34.96	31.48	5.11	30.30	21.99	38.67	<0.001
	CET	35.04	4.11 ^ddd^	35.63	26.39	40.53	27.49	4.08 ^f^	26.36	22.77	36.37	31.31	8.91	31.26	26.80	47.51	
EE [kcal·min^−1^]	DSN	1.87	0.45 ^aa^	1.84	1.28	2.99	11.26	3.07	11.31	6.53	17.06	13.77	3.76 ^bbb^	14.06	7.99	21.03	<0.001
	CET	2.00	0.62 ^d^	1.93	1.17	3.79	11.00	3.65 ^f^	10.97	5.46	17.02	14.72	4.32 ^eee^	14.17	7.56	23.28	

* ANOVA Friedman test. ^a,c^ Significant difference between the load of DSN; ^a^ Difference between rest and AT; ^c^ Difference between AT and ML;^ bbb^ *p* < 0.001, ^aa,cc^ *p* < 0.01, ^a,c^ *p* < 0.05; ^d,e,f^ Significant difference between the load of CE; ^d^ Difference between rest and AT; ^e^ Difference between rest and ML; ^f^ Difference between AT and ML; ^ddd,eee,fff^ *p* < 0.001, ^dd,ff^ *p* < 0.01, ^d,f^ *p* < 0.05.

**Table 3:** Statistical comparisons of the cardiovascular values obtained during DSN and CET (n = 18).

Variables	Group	R					VAT					ML					*p* *
		Mean	±SD	M	MIN	MAX	Mean	±SD	M	MIN	MAX	Mean	±SD	M	MIN	MAX	Main Effect
HR [bpm]	DSN	73.67	12.62 ^a^	73.00	54.00	100.00	148.28	14.15 ^c^	150.50	120.00	174.00	166.22	9.32 ^bbb^	164.00	152.00	181.00	<0.001
	CET	73.28	11.31 ^d^	73.50	47.00	94.00	149.78	12.07 ^f^	149.00	128.00	171.00	170.89	10.95 ^eee^	172.00	154.00	189.00	
SV [mL]	DSN	71.94	22.34 ^aaa^	67.12	50.00	126.98	97.81	31.38	101.76	53.03	149.35	93.02	27.70 ^b^	97.49	62.63	139.39	<0.001
	CET	76.19	33.04 ^dd^	69.44	40.00	121.49	95.63	30.48	102.08	50.72	146.34	91.45	28.49	95.42	61.28	148.35	
CO [L·min^−1^]	DSN	5.17	1.25 ^aaa^	5.00	3.00	8.00	14.33	4.31	15.00	7.00	23.00	15.39	4.45 ^bbb^	16.50	8.00	23.00	<0.001
	CET	5.33	1.37 ^dd^	5.00	3.00	9.00	14.33	4.77	15.00	7.00	24.00	15.61	4.94 ^eee^	16.00	8.00	27.00	
VO_2_/HR [mL·bpm^−1^]	DSN	5.39	1.74 ^aaa^	4.94	3.67	10.00	15.32	4.44	15.98	8.39	22.38	16.40	4.53 ^bbb^	17.27	9.22	25.36	<0.001
	CET	5.93	3.17 ^d^	5.43	3.20	15.45	14.51	4.67 ^f^	14.96	7.89	21.24	16.49	4.86 ^eee^	16.05	8.39	25.44	

* ANOVA Friedman test. ^a,b,c^ Significant difference between the load of DSN; ^a^ Difference between rest and AT; ^b^ Difference between rest and ML; ^c^ Difference between AT and ML; ^aaa,bbb^ *p* < 0.001, ^a,b,c^ *p* < 0.05; ^d,f^ Significant difference between the load of CET; ^d^ Difference between rest and AT; ^f^ Difference between AT and ML; ^eee^ *p* < 0.001, ^dd^ *p* < 0.01, ^d,f^ *p* < 0.05.

**Table 4:** Gender comparison of the respiratory and metabolic values obtained during DSN and CET by women (n = 7) and men (n = 11).

Variables	Group	R					VAT					ML				
		Women		Men		*p* *	Women		Men		*p* *	Women		Men		*p* *
		Mean	±SD	Mean	±SD		Mean	±SD	Mean	±SD		Mean	±SD	Mean	±SD	
V_E_ [L·min^−1^]	DSN	10.47	3.60	11.73	3.24	0.211	43.19	11.03	72.78	12.72	0.001	58.20	11.64	108.22	22.70	0.001
	CET	10.26	2.92	12.96	2.36	0.044	40.66	10.04	72.69	10.31	0.001	75.46	11.26	116.75	37.69	0.006
VO_2_ [L·min^−1^]	DSN	0.34	0.04	0.41	0.11	0.151	1.73	0.25	2.57	0.52	0.003	1.97	0.19	3.18	0.52	0.001
	CET	0.38	0.14	0.43	0.14	0.285	1.55	0.55	2.58	0.55	0.002	2.10	0.58	3.28	0.73	0.003
VCO_2_ [L·min^−1^]	DSN	0.29	0.05	0.34	0.08	0.126	1.64	0.33	2.56	0.55	0.004	1.98	0.32	3.32	0.57	0.001
	CET	0.32	0.11	0.36	0.06	0.178	1.59	0.47	2.59	0.45	0.002	2.52	0.38	3.76	0.61	0.001
RER	DSN	0.84	0.06	0.85	0.06	0.375	0.94	0.10	1.00	0.06	0.285	1.00	0.11	1.05	0.10	0.724
	CET	0.83	0.05	0.86	0.09	0.211	1.05	0.13	1.02	0.11	0.596	1.24	0.23	1.16	0.15	0.536
VO_2_ [mL·min^−1·^kg^−1^]	DSN	5.71	1.76	5.36	1.63	0.375	29.14	5.21	34.18	7.93	0.126	33.14	5.01	41.91	6.06	0.008
	CET	6.57	3.10	5.64	1.43	0.659	25.86	8.32	34.09	7.60	0.035	35.29	9.41	43.36	9.88	0.151
EQO_2_	DSN	30.45	7.45	28.57	4.61	0.479	24.66	4.00	28.99	5.08	0.104	29.32	4.51	34.43	7.42	0.126
	CET	27.79	3.72	30.96	4.70	0.126	27.26	5.38	28.92	5.32	0.536	37.72	9.48	36.90	12.17	0.724
EQCO_2_	DSN	35.66	5.90	34.21	5.47	0.659	26.11	2.32	29.01	4.56	0.126	29.33	3.01	32.86	5.79	0.211
	CET	33.35	4.90	36.11	3.32	0.246	25.82	2.19	28.56	4.72	0.328	30.18	3.91	32.02	11.15	0.375
EE [kcal·min^−1^]	DSN	1.66	0.22	2.01	0.51	0.126	8.62	1.34	12.95	2.65	0.002	9.96	1.06	16.20	2.57	0.001
	CET	1.84	0.68	2.10	0.60	0.246	7.85	2.69	13.01	2.62	0.002	11.04	2.64	17.06	3.46	0.001

*p* * Mann–Whitney U test.

**Table 5:** Gender comparison of the cardiovascular values obtained during DSN and CET by women (n = 7) and men (n = 11).

Variables	Group	R					VAT					ML				
		Women		Men		*p* *	Women		Men		*p* *	Women		Men		*p* *
		Mean	±SD	Mean	±SD		Mean	±SD	Mean	±SD		Mean	±SD	Mean	±SD	
HR [bpm]	DSN	79.00	16.10	70.27	9.08	0.151	156.57	13.73	143.00	12.18	0.056	170.00	11.78	163.82	6.93	0.328
	CET	77.86	8.80	70.36	12.13	0.179	152.71	15.85	147.91	9.32	0.479	172.71	12.84	169.73	10.05	0.479
SV [mL]	DSN	54.79	6.79	82.86	22.00	0.001	64.68	12.32	118.89	18.06	0.001	63.17	11.75	112.01	14.18	0.001
	CET	61.09	18.99	85.80	37.12	0.044	63.15	12.15	116.29	16.83	0.001	62.68	13.54	109.76	17.93	0.001
CO [L·min^−1^]	DSN	4.29	0.76	5.73	1.19	0.015	10.14	2.12	17.00	2.97	0.001	10.71	1.89	18.36	2.54	0.001
	CET	4.71	1.38	5.73	1.27	0.151	9.71	2.56	17.27	3.20	0.001	10.86	2.61	18.64	3.38	0.001
VO_2_/HR [mL·bpm^−1^]	DSN	4.40	0.68	6.02	1.93	0.027	11.17	2.12	17.96	3.33	0.001	11.69	1.63	19.41	2.80	0.001
	CET	4.94	1.94	6.56	3.70	0.211	10.06	3.08	17.34	2.94	0.001	12.17	3.32	19.24	3.49	0.001

*p* * Mann–Whitney U test.

**Figure A1:**
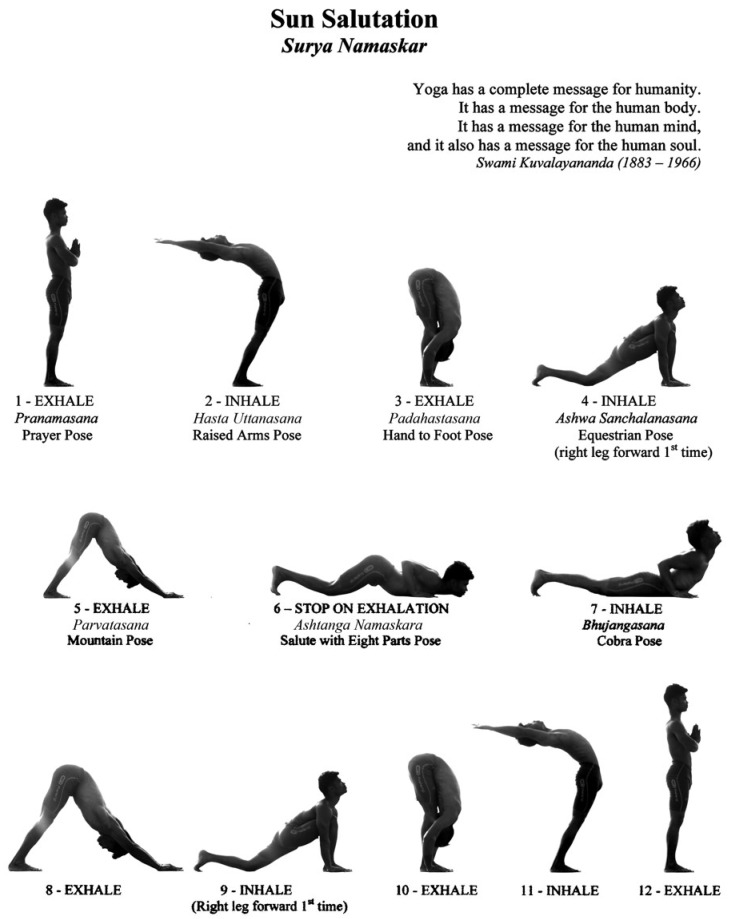
Sequence of asanas in the Rishikesh Series of Sun Salutations (Suryanamaskar).

## Data Availability

The data presented in this study are available on request from the corresponding author. The data are not publicly available due to its sensitivity and the privacy of the study participants.

## References

[1] Brouha L., Health C.W., Graybiel A. (1943). Step test simple method of measuring physical fitness for hard muscular work in adult men. Rev. Can. Biol..

[2] Hetzler R.K., Vogelpohl R.E., Stickley C.D., Kuramoto A.N., Delaura M.R., Kimura I.F. (2010). Development of a modified Margaria-Kalamen anaerobic power test for American football athletes. J. Strength. Cond. Res..

[3] Bjerregaard P., Ottendahl C.B., Jørgensen M.E. (2021). Hand grip strength and chair stand test amongst Greenlandic Inuit: Reference values and international comparisons. Int. J. Circumpolar Health.

[4] Robles-Romero J.M., Fernández-Ozcorta E.J., Gavala-González J., Romero-Martín M., Gómez-Salgado J., Ruiz-Frutos C. (2019). Anthropometric Measures as Predictive Indicators of Metabolic Risk in a Population of “Holy Week Costaleros”. Int. J. Environ. Res. Public Health.

[5] Bui H.T., Farinas M.I., Fortin A.M., Comtois A.S., Leone M. (2015). Comparison and analysis of three different methods to evaluate vertical jump height. Clin. Physiol. Funct. Imaging..

[6] Ayán-Pérez C., Cancela-Carral J.M., Lago-Ballesteros J., Martínez-Lemos I. (2017). Reliability of Sargent Jump Test in 4- to 5-Year-Old Children. Percept. Mot. Skills.

[7] Kaufmann S., Hoos O., Beck A., Fueller F., Latzel R., Beneke R. (2021). The Metabolic Relevance of Type of Locomotion in Anaerobic Testing: Bosco Continuous Jumping Test Versus Wingate Anaerobic Test of the Same Duration. Int. J. Sports Physiol. Perform..

[8] Enright P.L. (2003). The six-minute walk test. Respir. Care.

[9] Geiger R., Strasak A., Treml B., Gasser K., Kleinsasser A., Fischer V., Geiger H., Loeckinger A., Stein J.I. (2007). Six-minute walk test in children and adolescents. J. Pediatr..

[10] Cooper K.H. (1968). A Means of Assessing Maximal Oxygen Intake Correlation Between Field and Treadmill Testing. JAMA.

[11] Bunc V. (1994). A simple method for estimating aerobic fitness. Ergonomics.

[12] De Andrade V.L., Pereira Santiago P.R., Kalva Filho C.A., Zapaterra Campos E., Papoti M. (2016). Reproducibility of Running Anaerobic Sprint Test for soccer players. J. Sports Med. Phys. Fit..

[13] Stankiewicz B., Cieślicka M., Kujawski S., Piskorska E., Kowalik T., Korycka J., Skarpańska-Stejnborn A. (2021). Effects of antioxidant supplementation on oxidative stress balance in young footballers- a randomized double-blind trial. J. Int. Soc. Sports Nutr..

[14] Bar-Or O. A new anaerobic capacity characteristics and application. Proceedings of the 21st World Congress in Sports Medicine.

[15] Mullerpatan R.P., Agarwal B.M., Shetty T., Nehete G.R., Narasipura O.S. (2019). Kinematics of Suryanamaskar Using Three-Dimensional Motion Capture. Int. J. Yoga.

[16] Stec K. (2012). Dynamic Suryanamaskar—Sun Salutations.

[17] Satyananda S.S. (2008). Asana Pranayama Mudra Bandha.

[18] Ramaswami S. (2005). The Complete Book of Vinyasa Yoga.

[19] Durgananda S., Sivasananda S., Kailasananda S. (2018). Yoga, Your Home Practice Companion.

[20] Vivekananda K. (1977). Yoga: Asanas Pranayama Mudras Kriyas.

[21] Ni M., Mooney K., Balachandran A., Richards L., Harriell K., Signorile J.F. (2014). Muscle utilization patterns vary by skill levels of the practitioners across specific yoga poses (asanas). Complement. Ther. Med..

[22] Nidhi R., Padmalatha V., Nagarathna R., Ram A. (2012). Effect of a yoga program on glucose metabolism and blood lipid levels in adolescent girls with polycystic ovary syndrome. Int. J. Gynaecol. Obstet..

[23] Pal A., Srivastava N., Tiwari S., Verma N.S., Narain V.S., Agrawal G.G., Natu S.M., Kumar K. (2011). Effect of yogic practices on lipid profile and body fat composition in patients of coronary artery disease. Complement. Ther. Med..

[24] Stec K., Pilis W. (2017). Effects of dynamic suryanamaskar practice on serum lipid profile of Indian Students. Proceedings of the 4th International Multidisciplinary Scientific Conference on Social Sciences & Arts SGEM 2017, Albena, Bulgaria, 24–30 August 2017.

[25] Satyananda S.S. (2009). A Systematic Course in the Ancient Tantric Techniques of Yoga and Kriya.

[26] Sinha B., Sinha T.D. (2014). Effect of 11 months of yoga training on cardiorespiratory responses during the actual practice of Surya Namaskar. Int. J. Yoga.

[27] Bhutkar P.M., Bhutkar M.V., Taware G.B., Doijad V., Doddamani B.R. (2008). Effect of Suryanamaskar Practice on Cardio-respiratory Fitness Parameters: A Pilot Study. Al Ameen J. Med. Sci..

[28] Bhutkar M.V., Bhutkar P.M., Taware G.B., Surdi A.D. (2011). How effective is sun salutation in improving muscle strength, general body endurance and body composition?. Asian J. Sports Med..

[29] Chaya M.S., Kurpad A.V., Nagendra H.R., Nagarathna R. (2006). The effect of long-term combined yoga practice on the basal metabolic rate of healthy adults. BMC Compl. Altern. Med..

[30] Chatterjee S., Mondal S. (2014). Effect of regular yogic training on growth hormone and dehydroepiandrosterone sulfate as an endocrine marker of aging. Evid. Based Complement. Altern. Med..

[31] Paikkatt B., Singh A.R., Singh P.K., Jahan M., Ranjan J.K. (2015). Efficacy of Yoga therapy for the management of psychopathology of patients having chronic schizophrenia. Indian J. Psychiatr..

[32] Bhavanani A.B., Udupa K., Ravindra P.N. (2011). A comparative study of slow and fast suryanamaskar on physiological function. Int. J. Yoga.

[33] Sinha B., Sinha T.D., Pathak A., Tomer O.S. (2013). Comparison of cardiorespiratory responses between Surya Namaskar and bicycle exercise at similar energy expenditure level. Indian J. Physiol. Pharmacol..

[34] Stec K., Pilis K., Pilis W., Michalski C. (2016). Individual metabolic differences during dynamic suryanamaskar. Pol. J. Sport Med..

[35] Iyengar B.K.S. (1966). Light on Yoga.

[36] Borg G.A.V. (1982). Psychophysical bases of perceived exertion. Med. Sci. Sports Exerc..

[37] Sales M.M., Sousa C.V., Aguiar S.S., Knechtle B., Nikolaidis P.T., Alves P.M., Simões H.G. (2019). An integrative perspective of the anaerobic threshold. Physiol. Behav..

[38] Fujii T.K., Phillips B.J. (2003). Quick Review: The Metabolic Cart. Internet J. Intern. Med..

[39] Stringer W.W., Hansen J.E., Wasserman K. (1997). Cardiac output estimated noninvasively from oxygen uptake during exercise. J. Appl. Physiol..

[40] Hagins M., Moore W., Rundle A. (2007). Does practicing Hatha yoga satisfy recommendations for intensity of physical activity which improves and maintains health and cardiovascular fitness?. BMC Complement. Altern. Med..

[41] Mody B.S. (2011). Acute effects of Surya Namaskar on the cardiovascular & metabolic system. J. Bodyw. Mov. Ther..

[42] Larson-Meyer D.E. (2016). A Systematic Review of the Energy Cost and Metabolic Intensity of Yoga. Med. Sci. Sports. Exerc..

[43] Astrand P.O., Rodahl K. (1986). Textbook of Work Physiology: Physiological Bases of Exercise.

[44] Binder R.K., Wonisch M., Corra U., Sohen-Solal A., Vanhees L., Saner H., Schmid J.P. (2008). Methodological approach to the first and second lactate threshold in incremental cardiopulmonary exercise testing. Eur. J. Cardiovasc. Prev. Rehabil..

[45] Kenney W.L., Wilmore J., Costill D. (2012). Physiology of Sport and Exercise.

[46] Christophe C., Chodek-Hingray A., Pruna A., Bruntz J.F., Chometon F., Groben L., Huttin O., Aliot E., Juilliere Y., Selton-Suty C. (2009). Corrélation entre function atriale et capacité fonctionnelle chez les sportifs de haut niveau. Ann. Cardiol. Angeiol..

[47] Jakhotia K.A., Shimpi A.S., Rairikar S.A., Mhendale P., Hatekar R., Shyam A., Sancheti P.K. (2015). Suryanamaskar: An equivalent approach towards management of physical fitness in obese females. Int. J. Yoga.

[48] Chandla S.S., Sood S., Dogra R., Das S., Shukla S.K., Gupta S. (2013). Effect of short-term practice of pranayamic breathing exercises on cognition, anxiety, general well-being and heart rate variability. J. Indian Med. Assoc..

[49] Agre S., Agrawal R., Ishrat S. (2021). Effect of Suryanamaskar on stress levels in SSC students. Indian J. Public Health Res. Dev..

[50] Hipparagi M., Gangadhar P. (2019). Suryanamaskar for human wellness. Int. J. Phys. Educ. Sports Health.

